# Population structure and genetic diversity of *Mycobacterium tuberculosis* in Ecuador

**DOI:** 10.1038/s41598-020-62824-z

**Published:** 2020-04-10

**Authors:** Daniel Garzon-Chavez, Miguel Angel Garcia-Bereguiain, Carlos Mora-Pinargote, Juan Carlos Granda-Pardo, Margarita Leon-Benitez, Greta Franco-Sotomayor, Gabriel Trueba, Jacobus H. de Waard

**Affiliations:** 1 Instituto de Microbiología, Colegio de Ciencias Biológicas y Ambientales, Universidad San Francisco de Quito, Quito, Ecuador; 2 Instituto Nacional de Salud Pública e Investigación Leopoldo Izquieta Pérez, Guayaquil, Ecuador; 3 One Health Research Group. Universidad de las Américas, Quito, Ecuador; 4 Laboratorio para Investigaciones Biomédicas. Escuela Superior Politécnica del Litoral, Guayaquil, Ecuador; 5 Facultad de Ciencias Médicas. Universidad Católica Santiago de Guayaquil, Guayaquil, Ecuador; 6 Departamento de Tuberculosis, Servicio Autónomo Instituto de Biomedicina “Dr. Jacinto Convit”, Universidad Central de Venezuela, Caracas, Venezuela

**Keywords:** Microbiology, Clinical microbiology

## Abstract

Tuberculosis (TB) is a significant public health problem in Ecuador with an incidence of 43 per 100,000 inhabitants and an estimated multidrug-resistant-TB prevalence in all TB cases of 9%. Genotyping of *Mycobacterium tuberculosis* (MTBC) is important to understand regional transmission dynamics. This study aims to describe the main MTBC lineages and sublineages circulating in the country. A representative sample of 373 MTBC strains from 22 provinces of Ecuador, with data comprising geographic origin and drug susceptibility, were genotyped using 24 loci-MIRU-VNTR. For strains with an ambiguous sublineage designation, the lineage was confirmed by Regions of Difference analysis or by Whole Genome Sequencing. We show that lineage 4 is predominant in Ecuador (98.3% of the strains). Only 4 strains belong to lineages 2-sublineage Beijing and two strains to lineage 3-sublineage Delhi. Lineage 4 strains included sublineages LAM (45.7%), Haarlem (31.8%), S (13.1%), X (4.6%), Ghana (0.6%) and NEW (0.3%). The LAM sublineage showed the strongest association with antibiotic resistance. The X and S sublineages were found predominantly in the Coastal and the Andean regions respectively and the reason for the high prevalence of these strains in Ecuador should be addressed in future studies. Our database constitutes a tool for MIRU-VNTR pattern comparison of *M. tuberculosis* isolates for national and international epidemiologic studies and phylogenetic purposes.

## Introduction

*Mycobacterium tuberculosis* (MTBC) is the causative agent of tuberculosis (TB), an infectious disease that remains worldwide the first cause of death related to one single agent. The World Health Organization (WHO) estimated that 10 million people developed TB in 2017 and about 1.3 million died. Estimated Multi Drug-resistant (MDR) TB cases have increased to about 450,000 in the same year^1^. In this context, Ecuador (with a population of approximately 17 million), is considered a middle burden country with about 5,800 notified TB cases in 2017 (Incidence 43/100,000), with 480 TB deaths and 249 registered MDR-TB cases^2^. However, according to the 2018 WHO report, Ecuador may have a significant underdiagnosis problem; as WHO estimate is 7,200 TB cases with approximately 650 TB drug-resistant or MDR cases by year, corresponding to 9% of the country’s total TB cases^1^.

The ethnic makeup of Ecuador, a middle-income country with an estimated population of 17 million, is highly diverse. The urban population accounts for 60.43% of the total population. The ethnic composition of the population is 71.9% Mestizo, 6.1% European descendants, 6.8% Indigenous, 7.2% Afro-Ecuadorian, and 7.4% Montubian^3^. Indigenous people present heavy concentrations in the Andean region and the Amazon Basin. Monitoring of the genotypes of MTBC strains helps to understand the transmission dynamics of circulating clones in certain geographic regions or ethnic groups and can help to improve control and prevention programs^4^. Strain genotypes may play a role in disease outcome, variation in vaccine efficacy and the emergence of drug resistance. Moreover, major MTBC lineages are associated with patients’ country of origin. In the last 25 years, different methodologies have been developed for MTBC genotyping; IS6110-RFLP fingerprinting, spoligotyping and Mycobacterial Interspersed Repetitive Unit Variable Number of Tandem Repeat (MIRU-VNTR) typing. More recently, whole-genome sequencing (WGS) has been introduced as the most accurate method for studying molecular epidemiology of MTBC. However, WGS is still a challenging and expensive approach for developing countries like Ecuador. MIRU-VNTR is considered a gold standard for MTBC genotyping, showing an acceptable discriminatory power and reproducibility for studying the genetic diversity and biogeographical structure of MTBC at a local, regional or national level^5^.

Although the MTBC genetic diversity has been well described for some countries of the Americas, there is an important lack of information for others^6^, and there is little information on the genetic diversity of *M. tuberculosis* in Ecuador. Two reports showed a low prevalence of the Beijing family in the country^7,8^ and another study analyzed strains from the capital Quito showing a high prevalence of lineage 4^9^. Although this latter was a pioneer study in addressing the population structure of MTBC in Ecuador, it has limitations due to the limited sample size (104 isolates), drug resistance profile (all isolates were resistant to isoniazid, rifampicin or both) and location (all strains came from one single hospital in Quito). The present work aimed to study the genotypic diversity of strains of MTBC in Ecuador with a countrywide MTBC strain collection and compare our results with reports from other Latin-American countries.

## Materials and Methods

### Ethics statement

The study was approved by the ethics committee of University San Francisco de Quito (code 2017-023IN) and Instituto Nacional de Salud Pública e Investigación Leopoldo Izquieta Pérez” (INSPI), both certified by Ministry of Public Health from Ecuador following guidelines from Declaration of Helsinki. All samples were anonymized and no data of the patients were made available.

### MTBC isolates

A total of 373 isolates, one per patient, were selected for this study from the strain bank of the “Laboratorio de Referencia de Micobacterias” at the “Instituto Nacional de Salud Pública e Investigacion (INSPI) Leopoldo Izquieta Pérez” in Ecuador. Strains were provided by two public INSPI laboratories in Quito and Guayaquil, the only public laboratories in Ecuador that carry out mycobacterial culture and Guayaquil the only laboratory in Ecuador that performs drug resistance testing for the first- and second-line TB antibiotics. Consequently, MDR samples were usually much more likely to be collected by these laboratories. Strains from all provinces were included with exception of Imbabura and Carchi; no strains were available for these provinces, however, these provinces reported less than one percent of total national TB caseload. Ecuador comprises 4 distinct geographical regions: Andean, Costal, Amazonia and Galapagos Islands. Since we obtained only 1 strain from the Galapagos Islands we will refer to 3 geographical regions henceforth.

The strains included in this study were isolated in the years: 2013 (n = 41), 2014 (n = 121), 2015 (n = 179), and 2016 (n = 32) (Table 1). The sample size for each province was calculated using standard statistical methods^8^. Briefly, a cluster-randomized sampling method with an error = 0.05 and z = 1.96 was used to obtain a representative sample and in proportion with the number of reported smear-positive cases in 2013 and reported MDR case for each province. See Supplementary Table 1 for details of the strain distribution.

**Table 1:** Numbers and percentages of the sublineages found in Ecuador for specific geographic regions.

MTBC Sub Lineages	Costal region	Andes region	Amazon region	Total
LAM	133 (49.8%)	29 (36.3%)	8 (50%)	170 (45.6%)
Haarlem	88 (32.3%)	28 (35%)	4 (25%)	120 (32.2%)
S	29 (10.8%)	17 (21.3%)*	3 (18.8%)	49 (13.2%)
X	13 (5.2%)	2 (2.5%)	1 (6.3%)	16 (4.3%)
Beijing	1 (0.4%)	3 (3.8%)	—	4 (1.1%)
Ghana	2 (0.7%)	—	—	2 (0.6%)
Delhi	2 (0.7%)	—	—	2 (0.6%)
New	—	1 (1.3%)	—	1 (0.3%)
Lineage 4 without assignation	8 (2.2%)	—	1 (0.3%)	9 (2.4%)
Total	268 (73.4%)	80 (21.9%)	16 (4.4%)	373 (100%)

**Figure 1:**
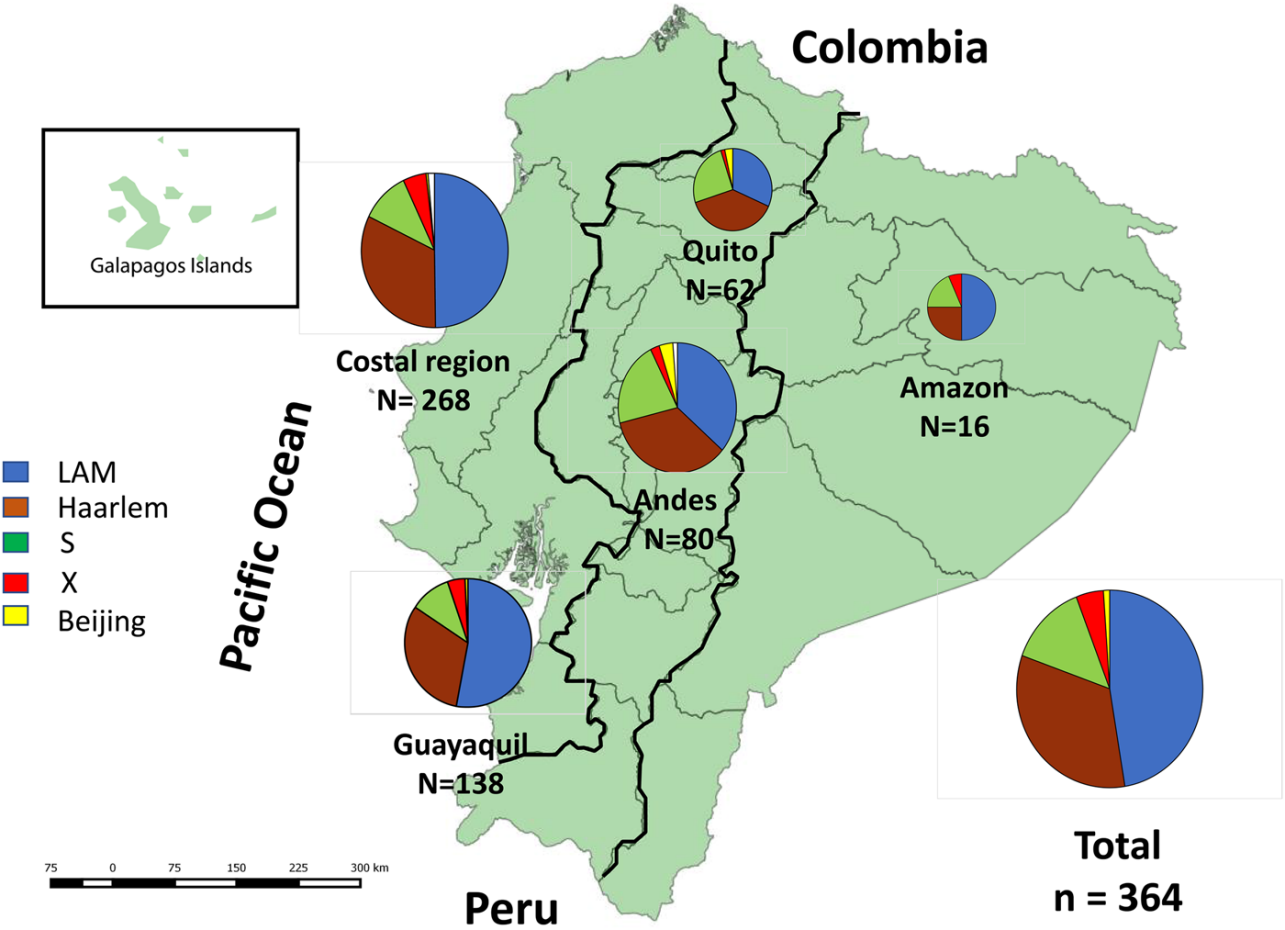
Distribution of the sublineages in Ecuador by geographic region and in the two principal cities of Ecuador. The map of the country shows the pie charts of the distribution of the major sublineages for the three main geographic regions, the coastal region including Guayaquil, the Andes region including Quito and the Amazon region. Also, in separated pie charts, the distribution of sublineages for the entire country and the two largest cities in Ecuador; Quito and Guayaquil (map was elaborated with sotfware Qgis 3.12; http://qgis.osgeo.org).

See also in Fig. 1. L2-Beijing, L3-Delhi; L4.1.1 or X; L4.1.2 or Haarlem; L4.3 or LAM; L4.4 or S; L4.1.3 or Ghana; L4.5 or NEW (*Prevalence significantly higher; p = 0.038).

### Drug susceptibility testing

Drug susceptibility testing for first-line drugs was performed using the proportion method of Canetti *et al*. on L–J medium with a minimal inhibitory concentrations of 40 µg/mL for RIF, 0.2 µg/mL for isoniazid, 4 µg/mL for streptomycin, 0.4 mg/mL for ethambutol and 200 µg/mL for pyrazinamide. The MTBC H37Rv strain was used as a control strain for drug susceptibility testing.

### DNA extraction

Several colonies from isolates grown in Ogawa*-*Kudoh medium were resuspended in 500 µl of TE (10 mM Tris pH 8.0 1 mM EDTA pH 8.0), boiled at 95° for 45 minutes, and centrifuged at 10,000 × *g* for 5 minutes. Supernatants were collected and stored at −80 °C until use.

### MIRU-VNTR genotyping

The 24-loci MIRU-VNTR typing was used in this study and a PCR for each of these loci was performed as has been previously described^10^. Products were analyzed by electrophoresis using 2% agarose gels, long runs of 3–4 hours and using 50 bp ladders as a band size reference. From the gel images, the corresponding MIRU-VNTR bands were interpreted, based on reference table indications, as copy numbers. To ratify the results, about 35% of the strains were analyzed two times.

### Regions of difference (RD) PCR genotyping

To confirm lineages and sublineages, especially for strains with an ambiguous MIRU-VNTR result, RD genotyping was performed for the chromosomal regions RD9, TbD1, RD4, RD5, RD10, RD12 RD 750, RD726, RD724, RD 239, RD105, RD702, RD711, IS1561, as previously described by Gagneux *et al*.^11^. Sanger sequencing was performed for the detection of the 7 bp deletion in the polyketide synthase locus (pKS1–15 RD) enabling the identification of Euro-American, Indo-Oceanic and Asian lineages^12–14^. The sequence analysis was carried out with the Geneious program (Biomatters); 253 of the 373 isolates (68%) were submitted to RD analysis including 220 strains representing all major and minor branches of the minimal spanning tree and 33 strains with a doubtful or ambiguous sublineage assignation.

### Whole genome sequencing (WGS)

WGS was carried out for 8 isolates with an ambiguous result for MIRU-VNTR and DR analysis Also, 3 Beijing strains were sequenced for confirmation and further studies. The Illumina Hiseq 4000 (Macrogen, South Korea) sequencer was used and Fastq files were analyzed using TGS-TB online platform available at https://gph.niid.go.jp/tgs-tb/, obtaining a coverage over 99% in all samples. A maximum-likelihood core genome phylogeny tree analysis was done with RAxML (Randomized Accelerated Maximum Likelihood) with 1000 X bootstrap test^15^. Whole-genome sequences reported in this study were deposited in GenBank under the accession numbers: VBWI00000000, VBWH00000000, VBVE00000000, VBVJ00000000, VBVK00000000, VBVI00000000, VBVD00000000, VBWK00000000, VBVG00000000, VBWJ00000000, VBVH00000000.

### Phylogenetic analysis

To analyze the phylogenic relationship between the strains we carried out a minimum spanning tree (MST) analysis using the MIRU-VNTR*plus* web application available at https://www.miru-vntrplus.org/MIRU/index.faces. We used a categorical algorithm including both MIRU-VNTR and Regions of Difference data; stepwise weighted distance (*D*_SW_) measurements were used when only MIRU-VNTR data was available^16^. The comparison was done with previously reported MIRU types; 186 strains representing all known lineages and sublineages and stored in the same database. Following the algorithm published by Allix-Béguec, the best match analysis with a cut off value of 0.16 and posterior confirmation by tree-based analysis^16^ was performed using the numerical code MTBCC15-9 based on 15 discriminatory loci and 9 auxiliary loci, for sublineage assignment^17^.

### Clonal complexes (CC) and clusters

Clonal Complexes were defined as strains with a maximum of two MIRU loci difference^18^. Cluster strains are strains with an identical MIRU-VNTR pattern.

## Results

All 373 strains included in this study generated interpretable MIRU-VNTR patterns and these were compared with the database available in the MIRU-VNTR plus platform (www.miru-vntrplus.org). Because of inconclusive results, WGS was carried out in 8 strains showing MTBC sublineages: Haarlem (2 strains), 1 S strain, 5 strains of sublineage X. Also, we confirmed with WGS the three Beijing strains present in our strain collection. The sequenced S strain belonged to sublineage 4.4.1.1. The results of WGS can be found in Supplementary Fig. 1. Mutations associated with drug resistance can be found in Supplementary Table 4.

Our analysis showed that lineage 4 is the most prevalent in Ecuador (n = 367 or 98.4%), and only 6 of 373 strains belonged to lineage 2-sublineage Beijing (n = 4,1.1%) and lineage 3-sublineage Delhi (n = 2/0.6%). The sublineage distribution of lineage 4 included: LAM (n = 170, 45.6%), Haarlem (n = 120, 31.2%) S (n = 49, 13.1%), X (n = 16, 4.3%), Ghana (n = 2, 0.6%), and New (n = 1, 0.3%). See Table 1. Additionally, 9 isolates (2.4%) belong to lineage 4 by MIRU-VNTR and RD analyses could not be associated with any sublineage. These strains were grouped in a minimum spanning tree analysis near the Haarlem (3), X (4) and S (2 strains) sublineages.

We found no statistical difference in the distribution of the TB sublineages LAM and Haarlem (Pearson *X*^2^ = 10.242, df = 8, *p* = 0.248) for Ecuadorian geographical regions. The S lineage, with 49 isolates, was predominantly found in the Andean region, especially in Quito, and in the Amazonia, both regions with twice the prevalence in comparison with the Coastal region. The X sublineage, with 16 isolates, was predominantly found in the Coastal region (13 isolates), especially in Guayaquil, and only 1 and 2 isolates in the Amazonia and the Andean regions respectively (Table 1). The Ghana and Delhi sublineages, both with two strains, were only found in the coastal region. In Fig. 1 we have visualized the distribution of MTBC genotypes for Ecuador and in Table 1 the details of the numbers and prevalence rates for the three main geographic regions of Ecuador are shown.

The sublineage distribution for the 2 largest cities in Ecuador, (Quito and Guayaquil, with about 2 million and 2.7 million inhabitants respectively) is shown in Table 2 and Fig. 1. There was a statistically significant difference (Pearson *X*^2^ = 13.237, df = 4, p = 0.009) in LAM prevalence in Quito and Guayaquil with 31.2% (n = 19) and 53.0% (n = 71) respectively. The prevalence of the S strains in Quito was twice that of Guayaquil, conversely, the prevalence of X strains in Guayaquil was double than that in Quito.

**Table 2:** MTBC lineages distribution for the two main cities in Ecuador, Quito, and Guayaquil.

MTBC sublineages	Guayaquil	Quito
LAM*	71 (53%)	19 (31.2%)
Haarlem	42 (31.3%)	23 (37.7%)
S*	14 (10.45%)	15 (24.6%)
X	6 (4.5%)	1 (1.6%)
Beijing	1 (0.8%)	2 (3.3%)
New		1 (1.6%))
Total	134 (35.9%)	61 (16.35%)

*Statistically significant difference in prevalence. (p < 0.05).

### Drug resistance profiles and MIRU-VNTR typing

Drug resistance profiles were available for 320 of the 373 MTBC isolates in this study (Table 3). Most of the strains were susceptible for all 4 first-line drugs (n = 171, 53.6%), 78 (24.5%) were MDR-TB, and 3 (0.9%) were polyresistant. Excluding Beijing, Delhi, and New because of the low number of isolates, MDR prevalence among lineages was only statistically different (Pearson *X*^2^ = 16.124, df 6, test, *p* = 0.013) for S strains with 10.3%, compared with LAM with 30.8%, X with 26.66% and Haarlem with 22.5%. For rifampicin resistance there was no statistically significant differences among the sublineages Haarlem (n = 5, 4.9%), LAM (n = 5, 3.4%), X (n = 1, 6.6%) and S (n = 1, 2.6%). A statistically significant differences (Pearson *X*^2^ = 16.608, df 9, *p* = 0.055) was found for isoniazid resistance in the sublineage LAM (n = 33, 22.6%) compared with sublineages Haarlem (n = 14, 13.7%), S (n = 5, 12.8%), and X (n = 2, 13.33%) (Table 3).

**Table 3:** Drug resistance profiles distribution among MTBC sublineages.

MTBC Lineages	Antibiotics			
	*Rifampicin*	*Isoniazid*	*MTBC (Isoniazid* + *rifampicin resistance)*	Susceptible to all drug tested
LAM*	5 (3.4%)	33 (22.6%)	45 (30.8%)	63 (43.1%)
Haarlem*	5 (4.9%)	14 (13.7%)	23 (22.5%)	60 (58.8%)
S*	1 (2.6%)	5 (12.8%)	4 (10.3%)	29 (74.4%)
X*	1 (6.6%)	2 (13.33%)	4 (26.66%)	8 (53.3%)
Beijing	1 (25%)	—	—	3 (75%)
Ghana	—	—	—	2 (100%)
Delhi	1 (50%)		1 (50%)	—
New	—	—	—	1 (100%)
Total	14 (4.5%)	54 (17.4%)	77 (24.48%)	166 (53.4%)

320 isolates with complete profiles were included; *indicates statistically significant differences (p < 0.05). For this analysis, we exclude the nine strains of the L4 lineages without a sublineage assignment.

### Clonal complexes and clusters

**Figure 2:**
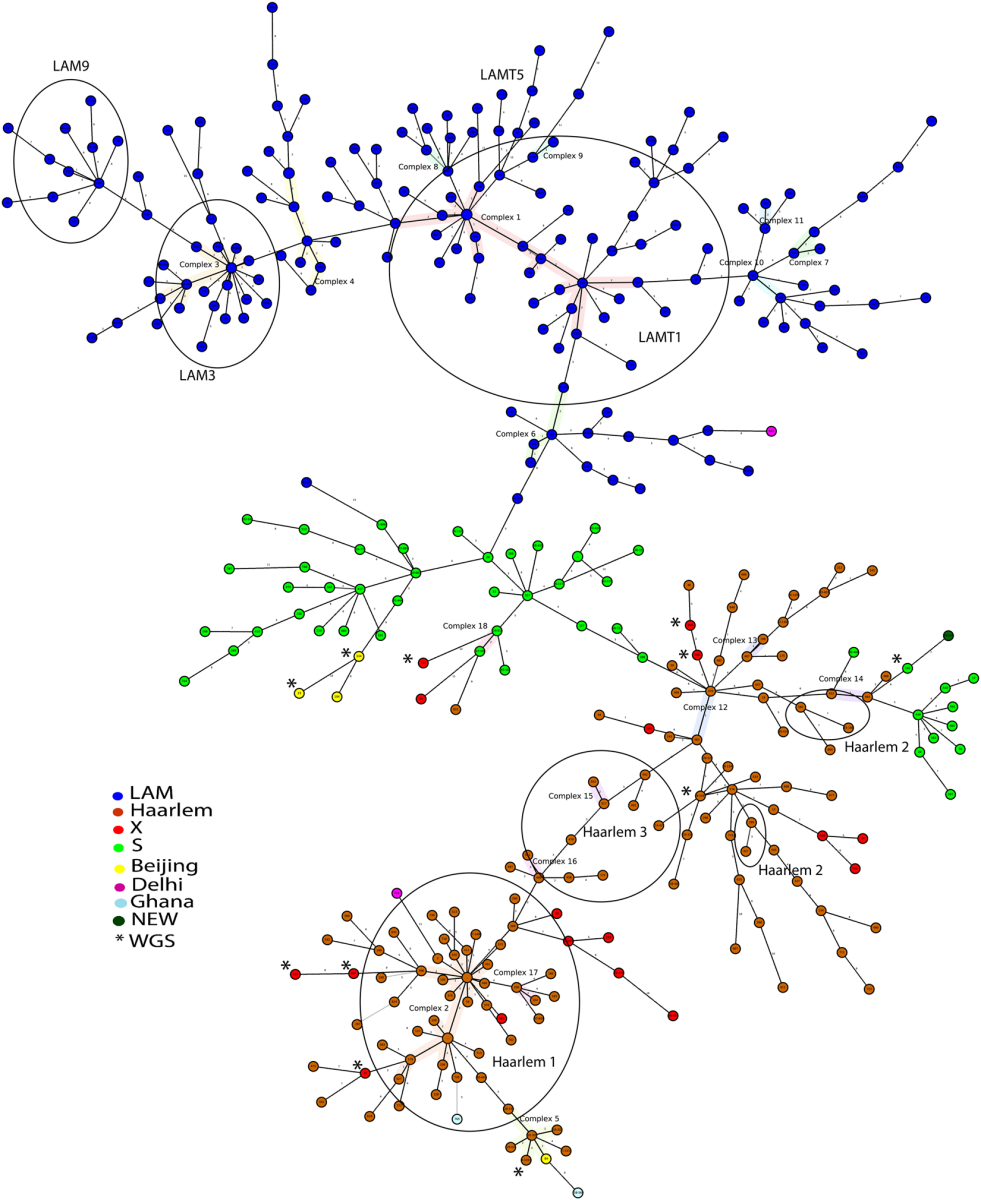
Minimum spanning tree of *Mycobacterium tuberculosis* isolates (n = 373) from Ecuador using MIRU-VNTR. The sublineage LAM is represented in blue, Haarlem in brown, S in green, X in red, Beijing in Yellow, Delhi in Violet, Ghana in white and New in Dark green. Nine strains of lineage 4 but without a defined sublineage were excluded. Isolates confirmed by Whole Genome Sequencing are indicated in circles with an asterisk.

Nineteen Clonal Complexes (CC), defined as strains with a maximum of two MIRU loci difference, were found encompassing 93 (24.9%) of the isolates. See Fig. 2 and Supplementary Table 2. Seven CC could be found only in Guayas province, comprising 52 of the 161 (32%) strains obtained from this Province. Other CCs were also found in Los Rios and El Oro provinces comprising 10 of the 44 (23%) isolates and 9 of the 21 isolates (43%) in these Provinces. This in contrast with the most densely populated province Pichincha were only 4 out of 61 strains (7%) formed part of a clonal complex. Moreover, 56 of the 171 LAM strains (33%) and 35 of the 118 Haarlem strains (30%) belonged to a CC, which may suggest no major differences in strain transmissibility. The biggest CC (comprising 22 LAM strains from Guayaquil) and another CC (comprising two S strains from Los Rios province) have been previously reported in Colombia^19,20^ (supplementary Table 2). Only six clusters (strains with an identical MIRU-VNTR pattern) compromising 16 strains were found, representing 4,3% of the strains (cluster index 2.6%) and an indication of potentially direct transmission. Five of these clusters were located in the Province of Guayas and one in Pichincha Province,

### Discriminatory power

The 24 MIRU-VNTR loci and their discriminatory power in Ecuador according to the three indexes are presented as Supplementary Table 3. The highest discriminatory power for the typing of the strains was observed in 12 allelic regions: 802(MIRU40), 2,163(Qub11), 424(Mtub02/MIRU42), 2,996(MIRU26), 960(MIRU10), 1,644(MIRU16), 1,955(Mtub21), 2401(Mtub30/MIRU47), 3,192(ETRE/MIRU31), 3,690(Mtub39/MIRU52), 4,052(QUB-26), 4,156 (QUB4156/MIRU53).

## Discussion

This investigation was undertaken to provide a nationwide analysis of the MTBC population structure in Ecuador. Only some countries in Latin America like Paraguay^21^, Chile^22–24^, Honduras^25^, Colombia^26–29^ and French Guiana^30^ have reported a nationwide analysis of the distribution of genotypes. Other countries with genotype reports like Peru^31–33^, Bolivia^34^, Venezuela^35,36^, Mexico^37^ and Brazil^38–41^ have concentrated their studies in densely populated urban areas, or strains with special characteristics like LAM Rio (Argentina^42^, Brazil^43–46^) and/or isolates with drug resistance (Argentina Peru and an earlier study from Ecuador^47–49^) or isolates from patients at high risk for drug resistance (Bolivia^34^). Also, most of the previous studies were done with spoligotyping or a combination of spoligotyping and MIRU-VNTR. We used a combination of MIRU-VNTR and DR typing. For sublineages difficult to resolve (for example the X sublineage), we used WGS for confirmation.

The Ecuadorian strains we analyzed in this study belonged mostly to lineage 4 which is dominant in all South American countries^6^ and it has been associated with the European colonization of Americas^50^. A low prevalence of two other lineages, namely 2 and 3 was found: lineage 2-Beijing has been detected in other countries in South America^6,30,33,34,51^ and was mostly linked to Chinese immigrants. Beijing has a high prevalence in Peru and Colombia but a low prevalence in Ecuador, which is in agreement with 4 previous reports^7–9,52^.

Sublineages LAM and Haarlem (Lineage 4) are predominant in Ecuador which is similar to the rest of South America^53^. Distribution of LAM was homogenous in Ecuador except in the Andean region where it has been replaced by sublineage S; in Quito with a prevalence of 24.6% and the rest of the Ecuadorian Andean region with 13%. In South America, the S strains have been detected only in Paraguay (9.5% prevalence), Venezuela, and Brazil (prevalence 2–4%)^6^. Data from Paraguay showed that the S strain is responsible for outbreaks^21^. The S strain (or L4.4 lineage), has a moderate global distribution, previously observed in parts of Asia and Africa and it is the most common strain in New Zealand, which accounts for 43% of New Zealand born L4 cases^54^. Whole-genome sequencing of one Ecuadorian S strain placed this strain into the L4.4.1.1 sublineage clade which is prevalent in indigenous populations of Polynesia and Canada^54^. It will be interesting to determine if the other Ecuadorian S strains also belong to this sublineage clade and infects preferentially a specific population in Ecuador and this needs further research.

In our hands, the X sublineage did not always resolve well by MIRU-VNTR and DR typing and we confirmed this sublineage by WGS in 6 strains. Lineage 4 sublineage X (L4.1.1) has a 4% prevalence in Ecuador which is higher than that of other South American countries except for Rio Grande in Brazil and Chile^22^. The L4.1.1 (X) sublineage mainly occurs in the Americas and lower proportions in few countries of southern Africa, Asia, and Europe^53^. In Chile, the proportion of X lineage has been reported noticeably higher in certain provinces, in Iquique and Concepción (11.7% in both), as compared to Santiago the capital (1.6%)^24^. This difference in Chile can be explained because of immigration patterns, which have greatly increased in recent years from countries like Peru, Colombia or Ecuador, or a genetic predisposition that makes the population of these cities more susceptible to certain genotypes^55^. In Ecuador, the Coastal prevalence is twice as high as the Andean one. We also found one strain of the NEW sublineage (Sublineage L4.5); as far as we know this is the first description in South America^6^. This strain is frequent in China, Vietnam, and the Filipinas and present in Angola and the United States^53^. The migration of Ecuadorians to and from the United States is a possible explication for the presence of this strain in Ecuador. Only 2 isolates belonged to sublineage Delhi (lineage 3) which is found mainly in East Africa, the Middle East, and India.

### Comparison of genotypes with another study from ecuador

A previous study that included 104 isolates from 1 hospital in Quito described six genotypes; LAM (33.7%) and Ghana (30.8%), S sublineage (5.8%), Cameroon (3.8%), Haarlem (3.8%), and Beijing (1.9%)^9^. Our study shows some discrepancies: they found 3.8% of Haarlem while we showed 37.7%; they found 30.8% of Ghana (mostly MDR), while we found 3.8% prevalence (non MDR); they found 3.8% prevalence of Cameroon sub-lineage which was absent in our study. Important differences among the studied populations can explain these differences. While we include 375 isolates from all over the country with a prevalence of drug-resistant strains of approximately 25%, the other study consisted of 104 strains, all drug-resistant isolates from a single hospital in Quito, Pichincha. This is a private hospital serving a specific population, introducing another, social and economic, bias. Our strains came from the INSPI institute serving the most needed of our population.

### Clonal complexes and their distribution in ecuador, colombia and peru

The minimum spanning tree analysis distinguished 19 clonal complexes made up of 2 to 22 strains (Fig. 2 and supplementary Table 2). Regarding the distribution of the clonal complexes (CC), only in 3 out of 18 of the CC were absent in Quito (Pichincha province) or Guayaquil (Guayas province), probably because these cities are important migration centers^56^ and migration plays an important role in the dissemination of sub-lineages. The largest CC comprised 22 LAM strains and some Colombian strains from Cali, Bogota, and Cauca^19,20^. The CC comprising 2 S sublineage strains from Los Rios province in Ecuador encompasses strains found in Colombia^19,20^. We did not find any clustering with Peruvian clonal complexes^33,58^. These differences may reflect differences in migration patterns. According to the United Nations, during the period that our MTBC strains were collected, approximately 170,000 Colombians were migrating to Ecuador while the number of Peruvian immigrants for the same period was around 15,000^57,59^. The proportion of clustering (Clonal Complexes) in the study area was 19.1%, and the recent transmission rate (cluster strains) was 2.6%. (Supplementary Table 2) Moreover, spatial analysis of the Ecuadorian strains showed that strains forming larger clusters had the characteristics of regional aggregation. The clustering rate, an indicator of recent TB transmission, in our study was relatively low. For example, a study conducted in London reported that 46% of the strains were clustered and the estimated proportion attributable to the recent transmission was 34%^59^. In the Netherlands, 46% of strains were found in clusters with identical RFLPs, and 35% were attributed to active transmission^60^. A low clustering rate has also been found in a previous study, in Quito, Ecuador with only 2 out of 104 isolates grouped to form one cluster^9^. Perhaps the cases in Ecuador arose mainly from the activation of the previous infection as it also has been suggested to explain the low clustering rate of strains from China^61^, due to the higher prevalence of latent infection in the Ecuadorian and Chinese population in comparison with the Dutch or English population. However, no data are available that reflect the prevalence of latent infection in Ecuador that can support this hypothesis. The tuberculin tests were only recently (the year 2017), incorporated in the National Tuberculosis Control Program in Ecuador as an auxiliary test, especially for the diagnosis of TB in children and immunosuppressed patients.

## Conclusions

This is the most comprehensive overview of the genetic diversity of *M. tuberculosis* in Ecuador to date and it is the first report of Delhi and New strains in South America. The MTBC population in Ecuador seems to have a limited diversity of sublineages among which LAM, Haarlem, and S are the most prevalent. The S genotype is an important lineage in the Andean and Amazonia regions, which may reflect an adaptation to the local population. Ongoing Venezuelan immigration, more than 4.5 million Venezuelans have left their country in the last 4 years and around 400.000 are living actually in Ecuador^62^, is likely to have implications for the pattern of tuberculosis genotypes in the future. The current baseline study provides information for future studies and our database constitutes a tool for MIRU-VNTR pattern comparison of *M. tuberculosis* isolates for national and international epidemiologic studies and phylogenetic purposes.

### Limitation of this study

No complementary spoligotyping data of our strains are available to confirm lineages and sublineages in this study though we confirmed the phylogenic status of an important part of our strains using RD and WGS. Our study has a relatively high prevalence of MDR-MTBC isolates (25.7%) which has introduced a bias factor. MDR TB prevalence has been reported to be 9% in Ecuador^63^. Since MDR is positively associated with LAM genotypes this would let to an overestimation of the presence of LAM in our study. No analysis associating age, sex, HIV status, diabetes and other predisposition factors for tuberculosis with certain genotypes could be established as data of patients were not available respecting patient privacy. S strains were easily identified with MIRU-VNTR typing but because this sublineage is not very common; a single isolate of the S strains (n = 49) was sequenced, indicating that this strain belonged to L4.4.1.1. The other S strains of this study or a part should also be sequenced to confirm this lineage assignment. It is well known that VNTR types are homoplastic and a recent study clearly showed that the many VNTR “clades” were not monophyletic, belonging to several clades when examined by WGS. This demonstrates the unreliable nature of defining a sub-clade based on MIRU-VNTR alone^64^.

## Supplementary information

Supplementary information.

Supplementary information2.

Supplementary information3.

Supplementary information4.

Supplementary information5.

## Data Availability

Data from this study will be made fully available and without restriction upon request.
