# Supplementary material for: Population structure and genetic diversity of Mycobacterium tuberculosis in Ecuador
Source: Sci Rep. 2020 Apr 10;10:6237. doi: 10.1038/s41598-020-62824-z (PMC7148308; doi:10.1038/s41598-020-62824-z)
Supplement: Supplementary file 1 — Supplementary information. [file 41598_2020_62824_MOESM1_ESM.docx]

**Population structure and genetic diversity of *Mycobacterium tuberculosis* in Ecuador.**

Daniel Garzon-Chavez, Miguel Angel Garcia-Bereguiain, Carlos Mora-Pinargote, Juan Carlos Granda-Pardo, Margarita Leon-Benitez, Greta Franco-Sotomayor, Gabriel Trueba and Jacobus H. de Waard.

**Supplementary table 1**. Distribution by Province of TB cases and MDR cases in Ecuador in 2013 and the *M. tuberculosis* strains and MDR strains by Province that were genotyped in this study. The sample size for each province was calculated using standard statistical methods^8^. Briefly, a cluster-randomized sampling method was used to obtain a representative sample and in proportion with the number of reported smear positive cases in 2013 to 2016 for each province. (Minimal sample size in this table). Because the strain bank not always contained sufficient (viable) strains by Province to reach the necessary number of strains for analysis, some Provinces did not reach the necessary number. However a final calculus showed that no significant difference was found for the distribution of notified TB cases and notified MDR cases by province and the distribution of strains or MDR strains by Province typed with the MIRU-VNTR technique, selected for this study (Wilcoxon signed-rank test respectively p<0.000 and p=0.016).

**^≈^**Ecuador. Instituto Nacional de Estadistica y Censos. <https://www.ecuadorencifras.gob.ec/censo-de-poblacion-y-vivienda/> Last time accessed December 2019

* Vigilancia Epidemiológica Coordinacion Zonal 9. Situacion de la Tuberculosis en el Ecuador. Ministerio de Salud Publica del Ecuador (MSP), Estrategia Nacional de Tuberculosis; 2014.

https://www.google.com/url?sa=t&rct=j&q=&esrc=s&source=web&cd=4&ved=2ahUKEwitifinzePmAhUFwFkKHTxcBhgQFjADegQIBxAC&url=http%3A%2F%2Frepositorio.puce.edu.ec%2Fbitstream%2Fhandle%2F22000%2F10431%2FTESIS%2520CVRSTBP.pdf%3Fsequence%3D1%26isAllowed%3Dy&usg=AOvVaw3G6ztZD8uNdrkWzHEzEl4Z Last time accessed 1 January 2020

^∞^77 MDR strains and 3 poly-resistant strains.

**For the calculation of the distribution of MDR strains by province, data of the year 2018 had to be used as no previous data for the distribution of MDR by Province are available for the previous years. See also: Ministerio de Salud del Ecuador. Informe de Tuberculosis 2017 -2018. <https://www.salud.gob.ec/gacetas-tuberculosis/> Last time accessed December 2019

| **Provinces** | **Population**  **Census Ecuador 2010^≈^** | **Notified TB cases by Province 2013*** | **Prevalence / 100.000 inhabitants** | **% of the notified TB cases of Ecuador by Province** | **Minimun sample size (2013)** | **Genotyped TB cases by Province** | **% of the total of genotyped strains** | **Notified MDR cases**  **2018**** | **MDR cases genotyped by Province^∞^** |
| --- | --- | --- | --- | --- | --- | --- | --- | --- | --- |
| **Guayas** | 3840319 | 2877 | 74.92 | 53.67 | 164 | 161 | 43.16 | 75 | 48 |
| **El Oro** | 634481 | 290 | 45.71 | 5.41 | 17 | 21 | 5.63 | 9 | 4 |
| **Los Ríos** | 817676 | 391 | 47.82 | 7.29 | 23 | 44 | 11.8 | 15 | 10 |
| **Manabí** | 1436259 | 229 | 15.94 | 4.27 | 13 | 13 | 3.49 | 10 | 1 |
| **Pichincha** | 2723509 | 251 | 9.22 | 4.68 | 15 | 61 | 16.35 | 3 | 5 |
| **Santo Domingo** | 387229 | 175 | 45.19 | 3.26 | 10 | 7 | 1.88 | 5 | 3 |
| **Loja** | 473331 | 71 | 15 | 1.32 | 5 | 2 | 0.54 | 3 | 0 |
| **Azuay** | 753943 | 119 | 15.78 | 2.22 | 7 | 8 | 2.14 | 1 | 2 |
| **Esmeraldas** | 561605 | 217 | 38.64 | 4.05 | 13 | 14 | 3.75 | 3 | 3 |
| **Santa Elena** | 326215 | 80 | 24.52 | 1.49 | 5 | 9 | 2.41 | 1 | 1 |
| **Sucumbíos** | 186072 | 84 | 45.14 | 1.57 | 5 | 10 | 2.68 | 0 | 0 |
| **Cotopaxi** | 431243 | 73 | 16.93 | 1.36 | 5 | 2 | 0.54 | 1 | 0 |
| **Chimborazo** | 48498 | 101 | 208.26 | 1.88 | 6 | 2 | 0.54 | 0 | 1 |
| **Imbabura** | 419919 | 77 | 18.34 | 1.44 | 5 | 0 | 0 | 1 | 0 |
| **Cañar** | 240248 | 80 | 33.3 | 1.49 | 5 | 5 | 1.34 | 3 | 0 |
| **Tungurahua** | 530655 | 52 | 9.8 | 0.97 | 3 | 2 | 0.54 | 0 | 0 |
| **Orellana** | 140663 | 69 | 49.05 | 1.29 | 4 | 1 | 0.27 | 1 | 1 |
| **Morona** | 157551 | 17 | 10.79 | 0.32 | 1 | 1 | 0.27 | 0 | 0 |
| **Bolívar** | 193689 | 36 | 18.59 | 0.67 | 3 | 2 | 0.54 | 0 | 0 |
| **Napo** | 109514 | 32 | 29.22 | 0.6 | 2 | 3 | 0.8 | 0 | 0 |
| **Pastaza** | 89053 | 5 | 5.61 | 0.09 | 1 | 2 | 0.54 | 1 | 1 |
| **Zamora** | 97676 | 26 | 26.62 | 0.48 | 2 | 2 | 0.54 | 0 | 0 |
| **Galápagos** | 26576 | 3 | 11.29 | 0.06 | 1 | 1 | 0.27 | 1 | 0 |
| **Carchi** | 173410 | 6 | 3.46 | 0.11 | 1 | 0 | 0 | 1 | 0 |
| **Total** | **14,779,334** | **5361** | **36.2** | **100** | **316** | **373** | **100** | **134** | **80** |
