# Supplementary material for: Population structure and genetic diversity of Mycobacterium tuberculosis in Ecuador
Source: Sci Rep. 2020 Apr 10;10:6237. doi: 10.1038/s41598-020-62824-z (PMC7148308; doi:10.1038/s41598-020-62824-z)
Supplement: Supplementary file 2 — Supplementary information2. [file 41598_2020_62824_MOESM2_ESM.docx]

**Population structure and genetic diversity of *Mycobacterium tuberculosis* in Ecuador.**

Daniel Garzon-Chavez, Miguel Angel Garcia-Bereguiain, Carlos Mora-Pinargote, Juan Carlos Granda-Pardo, Margarita Leon-Benitez, Greta Franco-Sotomayor, Gabriel Trueba and Jacobus H. de Waard.

**Supplementary table 2.** MTBC clonal complexes in Ecuador, identified by 24 MIRU-VNTR typing with respectively 2*, 1** and 0*** loci difference between strains. See also the MST of the MTBC isolates from Ecuador in figure 2. N is number of strains present in a clonal complex and the number of strains in a specific Province. $ are the clonal Complexes also detected in Colombia in ^18, 19^. To determine the clustering rate we applied the following formula; (total number of clustered isolates – Number of clusters)/Total number of isolates multiplied by 100 (Easterbrook 2004 and Mao 2018). *(1. Easterbrook PJ, Gibson A, Murad S, Lamprecht D, Ives N, Ferguson A, et al. High Rates of Clustering of Strains Causing Tuberculosis in Harare , Zimbabwe : a Molecular Epidemiological Study. 2004;42(10):4536–44; 2. Mao X. Clustering and recent transmission of Mycobacterium tuberculosis in a Chinese population. 2018;323–30).*

| Clonal complex | Lineage  and clade | Strains  2 loci* (n) | Strains  1 locus**(n) | Cluster Strains  0 loci***(n) | Provinces*  (n) | Provinces** | Provinces** |
| --- | --- | --- | --- | --- | --- | --- | --- |
| 1^$^ | LAM T1 | 22 | 8 | 3 | Guayas (20) Orellana (1) Pichincha (1) | **1.A** Guayas (5) Orellana(1)  **1.B** Guayas (2) | Guayas (3) |
| 2 | Haarlem 1 | 17 | 5    3 | 3  2 | Guayas (14) Esmeraldas (2) Zamora (n=1) | **2.A** Guayas (3) Esmeraldas (1) Zamora(1)  **2.B** Guayas (2)  Esmeraldas(1) | Guayas(3)  Guayas(1) Esmeraldas (1) |
| 3 | LAM 3 | 10 | 5 | 3 | Guayas (8) Pichincha (1) Bolivar (1) | Guayas (5) | Guayas (3) |
| 4 | LAM 3 | 9 | 0 | 3 | El Oro (4) Morona Santiago (1) Azuay (1) Los Ríos (1) Sucumbios (1) Chimborazo (1). | - | El Oro (1) Chimborazo (1) Sucumbios (1) |
| 5 | Haarlem1 | 6 | 0 | 0 | Los Ríos (2) Santa Elena (1) Esmeraldas (1) Pichincha (2) | - | - |
| 6 | LAM T1 | 4 | 4 | 0 | Guayas (4) | Guayas (3) | - |
| 7 | LAM T1 | 3 | 0 | 0 | Los Ríos (3) | - | - |
| 8 | LAM | 2 | 0 | 0 | Guayas (2) | - | - |
| 9 | LAM T1 | 2 | 0 | 0 | Guayas (2) | - | - |
| 10 | LAM T1 | 2 | 2 | 0 | Los Ríos (1), Manabi (1) | Los Ríos (1), Manabi (1) | - |
| 11 | LAM T1 | 2 | 0 | 0 | El Oro (1), Pichincha (1) | - | - |
| 12 | Haarlem1-Haarlem3 | 2 | 2 | 0 | El Oro (1), Pichincha (1) | El Oro (1), Pichincha (1) | - |
| 13 | Haarlem1 | 2 | 0 | 0 | Santa Elena (1), Quito (1) | - | - |
| 14 | Haarlem | 2 | 2 | 0 | Cañar (1), Azuay (1) | Cañar (1), Azuay (1) | - |
| 15 | Haarlem3 | 2 | 0 | 0 | Azuay (1), Manabi (1) | - | - |
| 16 | Haarlem3 | 2 | 0 | 0 | Esmeraldas (1), El Oro (1) | - | - |
| 17 | Haarlem1 | 2 | 0 | 0 | Guayas (2) | - | - |
| 18^$^ | S | 2 | 0 | 0 | Los Ríos (2) | - | - |
| 19 | S |  |  | 2 | - | - | Pichincha (2) |
| Total | **Clustering rate %** | 93  (24,9%) | 31  (8,3%) | 16  (4,3%) |  |  |  |
