# Supplementary material for: Population structure and genetic diversity of Mycobacterium tuberculosis in Ecuador
Source: Sci Rep. 2020 Apr 10;10:6237. doi: 10.1038/s41598-020-62824-z (PMC7148308; doi:10.1038/s41598-020-62824-z)
Supplement: Supplementary file 3 — Supplementary information3. [file 41598_2020_62824_MOESM3_ESM.docx]

**Population structure and genetic diversity of *Mycobacterium tuberculosis* in Ecuador.**

Daniel Garzon-Chavez, Miguel Angel Garcia-Bereguiain, Carlos Mora-Pinargote, Juan Carlos Granda-Pardo, Margarita Leon-Benitez, Greta Franco-Sotomayor, Gabriel Trueba and Jacobus H. de Waard.

**Supplementary table 3.** MIRU-VNTR regions used in this study and its discriminatory power calculated with the HGDI, Shanon and Simpson index according to recommendation of Mokrousow 2017 (** MIRU locus with high discrimination power). ***(****Mokrousov I. Revisiting the Hunter Gaston discriminatory index : Note of caution and courses of change. Tuberculosis [Internet]. 2017;104(2017):20–3. Available from: http://dx.doi.org/10.1016/j.tube.2017.02.002).*

| **Index/MIRU VNTR regions** | **HGDI** | **Shanon** | **Simpson** |
| --- | --- | --- | --- |
| 154/ MIRU 2 | 0.4657 | 1.137 | 0.5343 |
| 42/Mtub04/MIRU 42** | 0.7395 | 2.14 | 0.2605 |
| 577/ETRC/MIRU 43 | 0.568 | 1.469 | 0.432 |
| 580/ETRD/MIRU 4 | 0.1478 | 0.5195 | 0.8526 |
| 820/MIRU 40** | 0.817 | 2.596 | 0.1852 |
| 960/MIRU 10* | 0.6965 | 1.962 | 0.3035 |
| 1644/MIRU 16* | 0.6349 | 1.728 | 0.3668 |
| 1955/Mtub21* | 0.6882 | 1.979 | 0.3136 |
| 2059/MIRU 20 | 0.2234 | 0.6574 | 0.7773 |
| 2163/Qub11** | 0.7852 | 2.466 | 0.2169 |
| 2165/ETR A | 0.5849 | 1.531 | 0.4166 |
| 2347/Mtub29/MIRU 46 | 0.2732 | 0.9392 | 0.7275 |
| 2401/Mtub30/MIRU 47* | 0.7168 | 1.942 | 0.285 |
| 2461/ETRB/MIRU 48 | 0.1754 | 0.6149 | 0.8246 |
| 2531/MIRU23 | 0.5646 | 1.442 | 0.4369 |
| 2687/MIRU24 | 0.4232 | 0.1709 | 0.9578 |
| 2996/MIRU 26** | 0.7392 | 2.286 | 0.2627 |
| 3007/MIRU 27 | 0.4797 | 1.258 | 0.5215 |
| 3171/Mtub34/MIRU 49 | 0.4797 | 1.258 | 0.5215 |
| 3192/ETRE/MIRU 31* | 0.6163 | 1.616 | 0.3854 |
| 3690/Mtub39/MIRU 52* | 0.6663 | 1.832 | 0.3354 |
| 4052/QUB-26** | 0.8245 | 2.813 | 0.1777 |
| 4156/QUB4156/MIRU 53* | 0.664 | 1.854 | 0.3381 |
| 4348/MIRU 39 | 0.3831 | 0.9964 | 0.618 |
