# Supplementary material for: Population structure and genetic diversity of Mycobacterium tuberculosis in Ecuador
Source: Sci Rep. 2020 Apr 10;10:6237. doi: 10.1038/s41598-020-62824-z (PMC7148308; doi:10.1038/s41598-020-62824-z)
Supplement: Supplementary file 4 — Supplementary information4. [file 41598_2020_62824_MOESM4_ESM.docx]

**Population structure and genetic diversity of *Mycobacterium tuberculosis* in Ecuador.**

Daniel Garzon-Chavez, Miguel Angel Garcia-Bereguiain, Carlos Mora-Pinargote, Juan Carlos Granda-Pardo, Margarita Leon-Benitez, Greta Franco-Sotomayor, Gabriel Trueba and Jacobus H. de Waard.

**Supplementary table 4.** Mutations and codon changes, as determined with WGS for drug resistance markers.

| **Accession number** | **Linage** | **Resistance** | **Mutation** |
| --- | --- | --- | --- |
| VBWI00000000 | X | Rifampicin | rpoC V483G |
|  |  | Isoniazid | KatG S315T |
| VBWH00000000 | X | Rifampicin | rpoB H445D |
|  |  | Isoniazid | KatG S315T |
|  |  | Ethambutol | embB M306I |
| VBVE00000000 | X | susceptible |  |
| VBVJ00000000 | X | susceptible |  |
| VBVK00000000 | X | Rifampicin | rpoB S450L |
|  |  | Isoniazid | KatG S315T |
| VBVI00000000 | Beijing | susceptible |  |
| VBVD00000000 | Beijing | susceptible |  |
| VBWK00000000 | Beijing | Rifampicin | rpoB D435V |
|  |  | Ethambutol | embB G406D |
|  |  | Fluoroquinolones | gyrA D94A |
| VBVG00000000 | Haarlem | susceptible |  |
| VBWJ00000000 | Haarlem | susceptible |  |
| VBVH00000000 | S | susceptible |  |
