# Supplementary material for: Population structure and genetic diversity of Mycobacterium tuberculosis in Ecuador
Source: Sci Rep. 2020 Apr 10;10:6237. doi: 10.1038/s41598-020-62824-z (PMC7148308; doi:10.1038/s41598-020-62824-z)

**Population structure and genetic diversity of *Mycobacterium tuberculosis* in Ecuador.**

Daniel Garzon-Chavez, Miguel Angel Garcia-Bereguiain, Carlos Mora-Pinargote, Juan Carlos Granda-Pardo, Margarita Leon-Benitez, Greta Franco-Sotomayor, Gabriel Trueba and Jacobus H. de Waard.

**Supplementary figure 1**. Phylogenetic tree based on the complete genomes (WGS) of 44 strains of 14 sublineages. Strains of Ecuador with the respective accession number are indicated in bold in the tree.


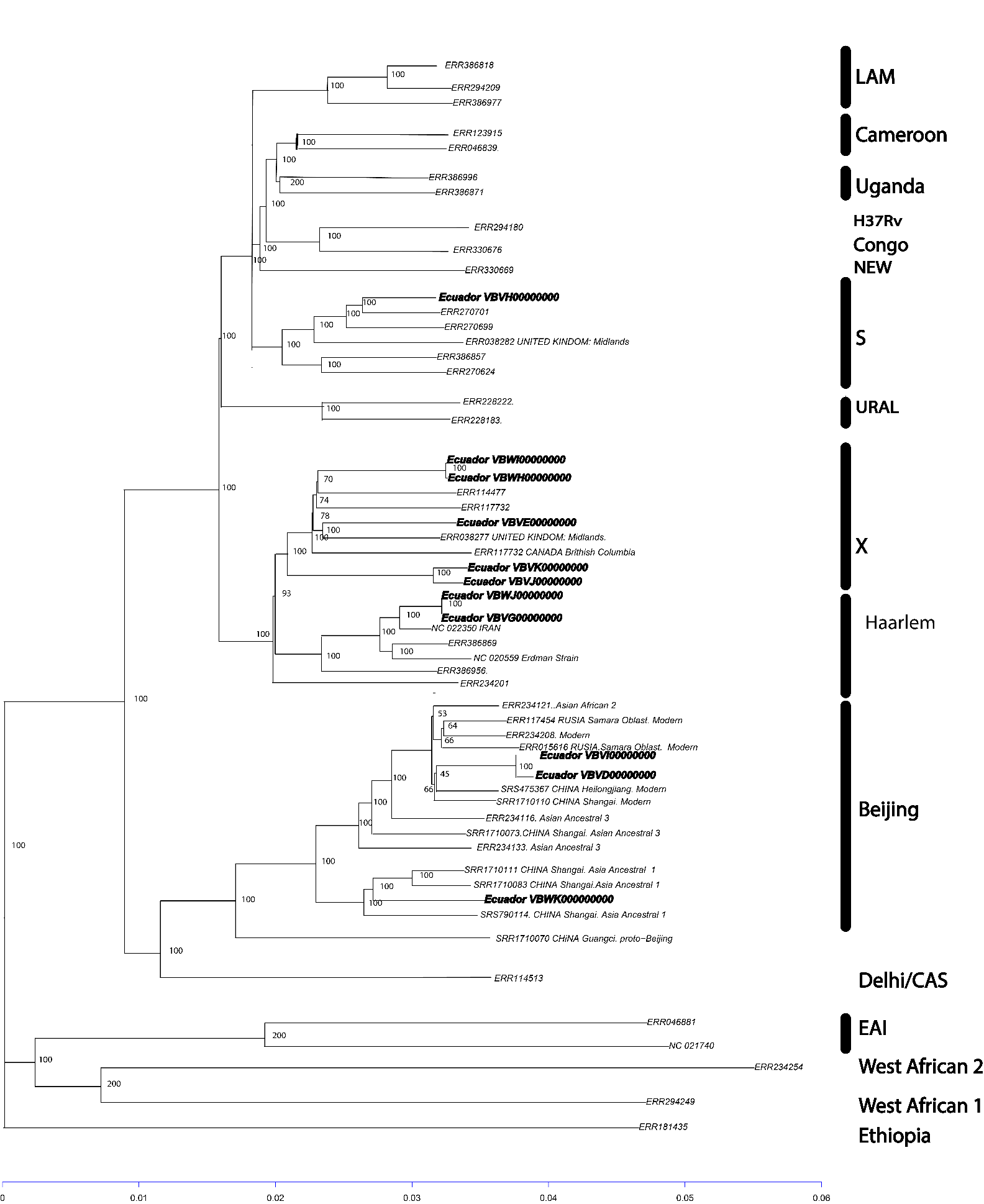

Supplement: Supplementary file 5 — Supplementary information5. [file 41598_2020_62824_MOESM5_ESM.docx]
